# “Prison didn’t change me, I have changed”: Narratives of change, self, and prison time

**DOI:** 10.1177/17488958211031336

**Published:** 2021-07-23

**Authors:** Katharina Maier, Rosemary Ricciardelli

**Affiliations:** University of Guelph, Canada; Memorial University of Newfoundland, Canada

**Keywords:** Change, identity, Imprisonment, self, time

## Abstract

Drawing on interview data with over 50 male former prisoners in Ontario, Canada, we examine male ex-prisoners’ narratives of change within prison settings. Specifically, we focus on how ex-prisoners talk about change to self and their persona, as they reflect back on both their pre-prison selves and the ways they believe prison changed them. We find that these ex-prisoners described prison as a time where they developed a more general sense of positive change. Ex-prisoners described how prison living made them “calmer,” “stronger,” and more “patient” overall. These descriptions stand in tension with the overall hostility of prison environments where prisoners are forced to focus on survival and basic well-being as they navigate the risks and threats of prison living. Overall, in this article, we seek to contribute to emerging discussions on positivity within prison settings, acknowledging that studying the more positive impacts of prison is a delicate yet important endeavor necessary to help better understand the experiential complexities of punishment.

A large body of literature documents how prisons operate as hostile, painful, and unsafe settings where fears for personal safety are pervasive and even omnipresent, and feelings of emotional and mental well-being are compromised (Hemmens and Marquart, 1999; Kellar and Wang, 2005; Wolff and Shi, 2001; Wolff et al., 2009; Wolff et al., 2007). Alongside these studies exists a small body of literature that recognizes, even within these hostile prison environments, prisoners develop more positive accounts of the impact prison can have on them (e.g. O’Donnell, 2014). In this article, we seek to contribute primarily to the latter body of work by discussing how self-identifying male former prisoners narrate self-change in the context of their incarceration. Specifically, we discuss prisoners’ narratives as they reflect on their sense of self and identity pre and post prison, and their interpretations of how and why one changes while incarcerated. In doing so, we seek to contribute to recent discussions that have grappled with the question of how positive change can ensue within the overall hostile environment of the prison (Crewe and Ievins, 2019). We argue that imprisonment can provide a space and time to reinvent or hold on to a narrative of self; for some, the narrative of self includes a new sense of identification built on promise and hope for what the future may hold, while for others the narrative is about staying true to self when faced with the complex and multifaceted vulnerabilities and challenges that shape prison living.

This article proceeds as follows: We first outline existing research on positivity within prison settings. Next, we review our methods and data. With the context established, we present the findings of our research in two parts: First, we explore how interviewees described their pre-prison selves and associated desire for self-change. Second, we examine how, when asked whether they felt they had changed in the course of their imprisonment, interviewees predominantly discussed positive forms of change to self, which include getting clean, becoming calmer and more patient, finding greater appreciation for family and loved ones, and recognizing they had “conned” themselves. Considering that prisons, fundamentally, are hostile environments, the discursive focus on positivity in participants’ narratives stands out as a rather surprising feature of prisoners’ description of prison life. As we have documented elsewhere (Maier and Ricciardelli, 2018), incarcerated men must spend considerable time thinking about how to best self-present to navigate feelings and actual experiences of threat and victimization. In other words, prison time may be more about survival and endurance (see also Wahidin, 2006) rather than personal reflection. Recognizing these realities and specifically the demands placed on male prisoners to present as tough, strong, and at times outwardly violent (see Maier and Ricciardelli, 2018), we add to existing scholarship by demonstrating how reinvention and positive self-change are created *alongside* existing demands of navigating hostility and risk potentiality. We show that positive self-change *can* and *does* happen for imprisoned men and reflect on the significance of acknowledging positivity as an important yet understudied facet of punishment. We discuss what about incarceration may generate positive self-change, pushing prisoners to “dig deep” and reinvent their self at the same time as they seek to overcome the challenges of incarceration.

By way of conclusion, we reflect on the seeming reluctance among prisons and punishment scholars to talk about the potential positive outcomes of imprisonment and ask how further understanding of positivity may change our thinking about prisons and punishment. Overall, we augment existing research by drawing attention to how prisons can function as places of positive narrative reinvention (Crewe and Ievins, 2019; Scott, 2010). Like other authors (Crewe and Ievins, 2019), we do not suggest that imprisonment is not painful, damaging, and destructive to prisoners’ and their loved ones’ lives, or that prisons are pleasant or helpful places. Rather, we seek to contribute to emerging conversations among punishment scholars about how we can make sense of and talk about prisoners’ accounts that entail more positive aspects of a person’s time in prison—accounts that emerge within and are shaped by overall hostile and painful environments.

## Prisoner narratives, sense of self, and institutional constraints

People’s identities and sense of self do not emerge in a vacuum but are constructed and reconstructed in the context of particular settings and relationships (Giddens, 1991). The prison is one situated context that constitutes or shapes people’s identities and sense of self. Goffman(1961: 168) in particular, in his seminal work “Asylums,” highlighted the influence of institutional power and constraints on a person’s self:

Each moral career, and behind this, each self, occurs within the confines of an institutional system, whether a social establishment such as a mental hospital or a complex of personal and professional relationships. The self, then, can be seen as something that resides in the arrangements prevailing in the social system for its members. The self in this sense is not a property of the person to whom it is attributed, but dwells rather in the pattern of social control that is exerted in connection with the person by himself and those around him. This special kind of institutional arrangement does not so much support the self as constitute it.

Goffman writes about the norms, organizational structures, and routines that define “total institutions,” like the prison, and the impactful role of institutionally located power in the construction of a person’s self. While Goffman leaves no doubt that authorship of the self is not the privilege of the individual (see also Scott, 2010: 213), it would be inaccurate to think of prisoners as completely powerless or repressed subjects regarding their own identity construction and presentation of self. Rather, as Scott’s (2010) analysis of Goffman’s work clarifies, prisoners are knowledgeable actors who, by practicing impression management, engage in projects of constructing and re-constructing the self.

Recently, scholars have started to pay closer attention to how, even within the “total” prison institution, “reinvention” of self among prisoners may happen. Drawing on Scott’s (2010) concept of the “reinventive institution,” Crewe and Ievins (2019) argue that while imprisonment evidently produces pains and hardships, we need to also consider prisoners’ competing narratives that suggest that imprisonment can lead to “narratives of reinvention” (p. 1) as well as more positive effects in prisoners’ lives. Specifically, Crewe and Ievins argue that prison can be a site of “moral action” (p. 14) insofar as prison may offer a *space* for reinvention to occur. Research by van Ginneken (2014) too shows that being imprisoned can facilitate a more positive reconstruction of prisoners’ identities. Together, these studies urge scholars to consider the nuances in prisoners’ adaptations to and experiences of incarceration. They also invite us to think more seriously about whether imprisonment can have a positive function in people’s lives, and if so, how scholars should talk about or make sense of these particular narratives without diminishing the pains and hardships that characterize, and in many cases dominate, prisoners’ descriptions of prison life.

Other scholars have noted that imprisonment can provide people with forms of temporary, tangible supports such as access to food and health care (Schinkel, 2014). Bucerius et al., (2020) explain how prison functions as a “temporary refuge” for women prisoners from the marginalization and material hardships they experienced on the outside; such “temporary refuge” can come in the form of temporary shelter and access to regular meals that individuals lacked in free society. For some, prison may even provide a space of protection from the harms and repeated victimization in the community that shape the lives of criminalized women at disproportionate levels.

These studies speak primarily to the destructive effects of shrinking welfare that precede incarceration and that, as a consequence, make the prison appear to some as a space of refuge and protection vis-à-vis the realities of life on the outside. In this article, we turn the focus inward, so to speak, by examining more closely the potentially positive or reinventive aspects of the prison experience on prisoners’ sense of self. Maruna et al. (2006) theorize that imprisonment can lead the way for “sudden identity transformation,” in part because prisoners, upon admission to custody, face a “crisis” of narrative (p. 161). Prisoners’ identity—who they are and how they want to (or can) be—is threatened by virtue of their incarceration. As Maruna et al. (2006) explain, the profound “identity crisis” can push prisoners to reinvent themselves (e.g. through religious conversion) and seek out new forms of self-identity, as a way to “make good,” find meaning in their punishment, seek forgiveness, or find hope for the future. We seek to add to the research and the work of others (Crewe and Ievins, 2019; Liebling et al., 2019; van Ginneken, 2014) that have paid closer attention to the role of the prison in shaping or (re-)inventing narratives of the self. We do so by highlighting the narratives of former prisoners who, in this study, reflected on their time spent federally in incarceration (see further below). We seek to highlight the complexities of prisoners’ descriptions of prison life, drawing attention to how the prison may give rise to more positive change at the same time as it demands constant vigilance, toughness, and displays of physical prowess. Our findings suggest that change in self is believed to emanate primarily from within prisoners’ selves—their will, desires, introspection, and insight—not the institution. Yet, even when change is perceived to emanate from within, institutional power is still at play in shaping prisoners’ sense of self (see Crewe and Ievins, 2019). In other words, we consider the broader context of a person’s incarceration in shaping the self. Thus, we discuss what elements of the prison or a person’s incarceration may contribute to the more positive narratives evident in our data, paying particular attention to how imprisonment disrupts pre-prison time and space, immersing prisoners into a new time and spatial settings that forces self-reflection.

## Methods

We drew the data for this article from a qualitative study of self-identifying male former prisoners in the province of Ontario, Canada. Because details of the study can be found elsewhere (see, for example, Maier and Ricciardelli, 2018; Ricciardelli, 2014), we keep our discussion of methods and methodological considerations here brief. In essence, the results are based on semi-structured, in-depth interviews with 56 male former prisoners, all of whom were incarcerated in at least one Canadian federal prison and, at the time of the interview, had been released into an urban location in Ontario, Canada. In Canada, people with a prison sentence of 2 years of more serve time in a federal penitentiary, while those sentenced to a maximum of 2 years less a day serve time in provincial/territorial prison institutions, with few exceptions. Participants in our study were sentenced between 2 years and life with parole and had been released into the community for anywhere from a few weeks to years (since their last experience of federal incarceration). The majority, almost 60%, had convictions that included violent crimes, such as murder and assault; 25% had non-violent, non-sexual convictions, such as drug trafficking; and 16% were convicted of sex-related offenses, such as making and distributing child pornography. Their ages ranged from 19 to 58 years, with a mean age of 37 years. The majority had previous experience in provincial institutions, and many had been to custody as youth. All participants served time in a maximum-security facility during their “reception” while awaiting their assessment and transfer to their “home” institution. Their “home” institutions varied in security classification (i.e. maximum, medium, minimum), which allowed us to ask participants about changes in their perspective and experience during their incarceration across institutions of diverse security classification.

Participant recruitment and interviewing occurred onsite at a day reporting center in Ontario. Center staff informed potential participants about the study and invited them to participate in interviews when the primary investigator was onsite. Interviews were conducted in person onsite in a private space away from center staff. Interviews included a wide range of questions focused on prisoners’ experiences of risk, safety, health care, relationships, and other facets of their imprisonment. After each interview was transcribed and coded, the authors analyzed the data using a semi-grounded constructed emergent theme approach with axil coding (Charmaz, 2006; Ricciardelli et al., 2010; Strauss and Corbin, 1990). We edited all excerpts from the data for speech fillers and anonymity. We used pseudonyms to protect participants’ identities.

## Reflecting on the pre-prison self

To learn how the prison might impact prisoners’ self, we first listened to, then analyzed, prisoners’ narratives of their pre-prison personas. We found that participants’ descriptions of their pre-prison selves in this study varied a fair bit. Some participants described themselves as a “good person” (Bill), while others held more negative conceptions, describing themselves as a “good person with bad intentions” (Jack) or a “bad man” (Max). However, no matter how they described their pre-prison selves, most, even those who described themselves as generally “good,” disclosed a rather negative part of their pre-prison identity, typically linked to a low sense of self-worth and feelings of regret. For example, some described themselves as people who were out of control prior to being federally sentenced, had made poor choices in the past, had acted irresponsibly and recklessly, or misused substances—all elements of self upon which imprisonment had pushed them to reflect on.

A consistent theme across participants, when talking about their pre-prison selves, was the notion that, at the time of their crime, their future was uncertain, as was their current situation. Nate, for example, described himself as somebody who was “out of control” and who

didn’t really care about anything. If I was to get shot, it would have been a relief, cuz I wasn’t doing anything good for anybody. More or less, I was just partying and committing crimes.

Participants like Nate, who was not unique in his desolate perspective on the value of his past and current life, felt entrapped in a lifestyle that he believed lacked any positive contributions to himself, the people around him, or society as a whole.

In a similar vein, participants also expressed, often with regret, that prior to their sentencing and imprisonment, they failed to recognize and accept help from people around them who they only later came to realize deeply cared about their well-being. A theme that arises across interviewees is that prior to their incarceration, many believed they were self-focused, almost, to their own detriment. Participants talked about their desire for instant gratification (i.e. getting high, wanting a particular lifestyle) or lack of regard at the time for those who genuinely loved them and cared for their well-being. In line with other studies (e.g. Maruna et al., 2006), being in prison, as participants stated, pushed them to reflect on their past lives and selves, which led them to consider how they envisioned their future selves. The realities of prison living (specifically the pains and deprivations; see Sykes, 1958) led prisoners to re-evaluate their pre-prison lives, relationships, and self which for many formed the basis for more profound self-change focused on who they wanted to be and how they envisioned their future. In particular, for interviewees in this study, being held away from their families appeared to increase feelings of identifying with and belonging to their loved ones on the outside.

## Narratives of positive self-change

Prisons are generally hostile environments where feelings of safety are limited. As some participants highlighted, living in the seemingly high-risk, low-safety environment of the prison forced some to focus primarily on their day-to-day (physical) existence. Participants talked about the need to act tough and display violence in prison “You have to fight, you have to fight even if you’re going to lose you have to fight [. . .]” (Sam). For several participants, these realities forced them to adopt new behaviors and forms of self-presentation, different from their pre-prison lives where, for example, Stan said he “never really had to fight people, this and that, shit like that [. . .].” As potentially hostile settings, prisons are arguably not conducive to positive self-change. For participants, much about being in prison was simply about surviving and staying physically and mentally healthy. Still, participants did feel they also underwent positive changes while in prison, occurring alongside constant negotiations of diverse vulnerabilities. The key self-changes acquired in prison, as reported by interviewees, included learning patience and being calmer; developing a drug-free narrative; realizing greater appreciation for loved ones; and gaining insight into their personal role in the path that led them to their incarceration. Positive self-change, thus, was not narrated as change from a pre-prison “bad” to a (post-)prison “good”; rather, participants talked about specific forms of self-change which they noticed during their time in prison.

### Patience and calmness

Interviewees spoke about how they acquired more patience and a stronger sense of calmness during their incarceration. The patience was more than simply learning to wait for meals, showers, yard time, mail, or other necessities and privileges; rather, participants talked about how they developed a new sense of patience which helped them to interpret life situations in different ways and which overall led them to adopt a new sense of appreciation for their own life. As Steve said,

Prison brought me patience, it made me look at life from a different angle. It made me know that there is a lot going on out here . . . Like out here, you can’t take it for granted . . . I think that’s what prison did for me . . . Patience and helping me appreciate the little simple things.

Like Steve, Jack also believed he had become more patient and “relaxed” in the course of his incarceration, which helped him appreciate the freedoms and privileges of living in the community. In prison, he learnt to look at life as something to value and cherish as he explained how prison

made me more relaxed . . . it made me look at life in a different way. Before [prison], I never used to pay such keen attention to life. But, since I come out I always appreciated life, but I appreciate it more now from being locked up . . . There’s nothing like your freedom . . . that’s a whole different mentality. That’s what’s happening to me right now. I really appreciate it, yeah.

The notion of becoming more “patient” or “relaxed” and the ways these changes in self shaped participants’ views and life perspectives were most commonly described using the adjective of “calm.” As Erwin explained, “it [prison] made me more calm than I was. It made me more calm and made me look at things through different perspectives.” Anthony, too, described how he learnt to “think about what you did and what you shouldn’t do. More or less, anticipate all the consequences, think before you act and all that. [In prison I was] more calm.” As evidenced in the words of these participants, prison was associated with change and transformation of self insofar as it taught participants certain qualities that they believed were useful in approaching and interpreting life situations on the outside. Patience and calmness, as the narratives show, were connected to feelings of inner strength and confidence; by learning to be patient, interviewees generated a general sense of calmness around their life and future that was only achievable by developing the inner strength to confirm that positioning—to make it possible to be patient and thus to calm down. As Scott, for example, explained,

In a way, I’m still the same person but more stronger now because I know I’ve been in prison. When you go to jail it clears your mind and makes you stronger so you could survive whatever situation you have to face in life.

Scott speaks of a clearing of mind, a result of him learning patience and calmness, which created space for reflection and new recognition for his inner strength. In essence, the experience of making it through the prison back to the outside gave participants a sense of confidence and achievement and a newfound sense of hope and recognition for who they were and could be in the future.

### Getting clean and healthy

In addition to patience and calmness, some participants spoke about making the decision to become clean from substance misuse. They said that prison had helped them in this endeavor insofar as it provided a space to “get clean” and to stop using drugs:

I lost so much I gave up so much and it was all feeling sorry for myself and you know what prison helped me, it’s a good thing I got the amount of time that I did cause if I got less time I would have still been doing drugs. I would have got out [and] nothing would have really changed. I kind of realize I have no reason to feel sorry for myself I have a pretty good life. (Nick)

Once I went to jail, I cleaned up a bit . . . [before prison I’d] Wake up, you’re sad in the morning for ten minutes, and you just go get high, right? Now it’s like, you can’t really run from them [your emotions] anymore, you have to deal with them, and that’s the experience I went through while I was in jail, right? . . . But I would just say, being in prison changed me lot . . . And that was the biggest eye-opener, I was seeing these people that are 50 years old that are still doing the same shit . . . It’s never too late, because every passing moment is another second to change it all around. And I do agree with that. But I just didn’t, that was my biggest deterrent. I didn’t want to be that 45-year-old guy in prison. I don’t want to be that guy. And having to deal with that every day, it was like, “wow, fuck.” And they support that. Like, “Yeah man, don’t be me, I fucked up, please learn from my mistakes.” (Mike)

Nick and Mike demonstrate through their words that they benefited from prison physically (e.g. their health improved by reducing drug consumption) and mentally (e.g. taking steps toward managing addiction). Nick credits the duration of his sentence to his “being clean,” while Mike attributes his ability to learn to manage his addiction to what he observed in prison—learning to manage his emotions and recognize the direction in which he wanted his life to proceed. Overcoming addiction, in any capacity and to any degree, is no small feat; thus, the reality that, for some participants, prison served as a site of rehabilitation and recovery, particularly for those who otherwise could likely not afford said private clinics, is an undeniable benefit of their incarceration experience. For these interviewees, the reinvention of self included a drug-free narrative, arguably foundational for pro-social living and re-entry.

### Appreciation for loved ones

Many participants talked about how in prison they learnt a new sense of appreciation for their loved ones. In the course of their imprisonment, they came to terms with the fact that imprisonment had caused them the loss of invaluable relationships, such as with their children. These participants recognized their own lack of appreciation that they had for their loved ones prior to their incarceration as the true value of these relationships only became obvious in prison. Interviewees said,

[it was the] realization of the people that I’ve hurt because of my incarcerations and just being mad enough to learn that I should have appreciated the people around me. I had kids before I went in so I was in for their life . . . (Brian)

Before I went in, I didn’t care what people had to say, if they were trying to help me or not, I just did my own thing. Then when I was sent to prison, you start to look for any kind of help, cuz you see other people in there doing life, or just repeat offenders and it’s like they don’t care about their freedom and all that. And then you just start to realize, you want your freedom, you don’t want to be in here. (Nate)

In the former excerpt, Brian’s words again demonstrate disappointment in self (e.g. “being mad enough to learn . . .”) in that he failed to appreciate those he loves; not only did he not take their support at the time, but his incarceration caused additional harm to his family considering that he missed out on irreplaceable years of his children’s lives. In the latter excerpt, Nate developed appreciation for his loved ones while in prison, although he also acknowledges that this appreciation was too long overdue. Overall, participants reflected on their relationships with loved ones, their own role in these relationships, and their desire to undo harm and re-build relationships after release.

### A new level of introspection

As a last element of self-change and transformation, participants also spoke about realizing in prison that they had “conned” themselves insofar as they had not taken responsibility for or really acknowledged their role in their own criminality prior to being sentenced and imprisoned. They learned to do so in prison by stating that they learnt to be true to themselves:

I always know who I am. My biggest problem before was that I conned myself. I conned other people but I conned myself. I justified the reason why I do things, like “oh it’s okay, because I’m sad,” or whatever. I do that to myself more than anyone. I conned myself more than I conned anyone else. And that’s what I realized, now that I address a lot more, like “really, is this going to be something that my children are going to be proud of at the end of ten years?” I think a lot more . . . I re-prioritized my entire life, basically, is what I’m trying to say. And I realized what is important, and what is not so important. (Mike)

I’m never going to be a millionaire or anything like this but I don’t need to be either . . . You know I didn’t finish high school but I’m not an idiot I again came to realize that I justified a lot of things to myself over the years. I think that’s relatively common justifying or trying to rationalize our behaviours. (Nick)

For Mike and Nick, prison then was a time for self-reflection, for learning what is important in life, and for becoming true to themselves. They, in prison, recognized their priorities and how they wanted to conduct themselves in relation to those they loved. In this context, participants shared feelings of remorse in reflecting on their pre-prison lives and the hurt they had caused to others. As Steven said,

I feel a lot of remorse. Even when I got sentenced I showed remorse . . . . The big picture was I hurt him, he’s hurt, this is his injuries and I’m a bad man. So I just have to take it as that. But as remorseful I feel, I felt very angry that I allowed myself to get [to that point].

As Steven’s words reveal, he felt dissatisfaction with himself based on his prior actions (the fact that he caused non-retractable harm to another person). Imprisonment, for Steven and others, provided a “hook” for putting an end to his prior choices and opportunity to change his future life and self. To summarize, participants’ narratives provide deeper insight into the forms of positive self-change and transformation that may emerge during a person’s incarceration. Following, we discuss more specifically how such self-reflection may occur within a largely hostile environment; in other words, we consider what it is about prison that may lead to the forms of introspection and self-change that stood out in participants’ narratives.

## Space, time, and change in self

Laced throughout each participant’s interview were discussions about the positive change in self that they believed they had experienced during their incarceration. For all participants, changing their life and self included being ready mentally to commit to “change.” Stewart, for example, explained that during his first federal sentence he did not change, but then the “second time around, I would say, ‘yeah, I grew up’.” Prisoners’ narratives of positive change were generally coached in the perception that prison, fundamentally, was negative—a terrible environment that was harmful to those incarcerated. As Brian put it,

Prison didn’t change me, I have changed but prison didn’t change me. So because prison hardly makes you a better person it more makes you a worse person, unless you can control yourself and realize things early in . . . You can't say being incarcerated changed you because being incarcerated I saw more violence inside then I’ve seen outside, it was more frequently inside [. . .]

While participants attributed their desire to change to self—their person, their will, and their inclinations—rather than to any tangible sorts of help and supports provided by the prison institution (e.g. prison programming), their narratives speak how imprisonment, by providing distinct space and time for reflection, functioned as a “hook” for self-change and the adoption of new narratives. Beyond attributing positive change to self, prisoners acknowledged that the realizations underpinning their positive self-developments did occur within prison, including within the hostile environment, as did the cementing of such developments.

For one, prison provided participants with space for reflection. For example, Ken whole-heartedly declared that, in prison, beyond knowing who he was and who he wanted to be, he also made “goals,” had “aspirations,” and remained “ambitious.” He said, “I benefited from my experience.” He did not attribute his positive self-change to prison living per se, explaining it is “not to say that if it wasn’t for that [prison] I wouldn’t have the mentality I have now,” but instead he described that “what I’m going to do with my life, [is] nothing negative . . . knowing that I have a second chance at life; I’m going to do what I can . . .” What prevails in his words, echoing others, is this idea that he gained *something* while inside. For Ken and others (see, for example, Mike’s narrative above), new aspirations emerged in response to realizing how their life would shape up if they continued down the path of their pre-prison life and wanting to avoid further hurt to their loved ones and themselves. Similarly, other participants, as evinced in the narratives below, focused on their own role in realizing they wanted to change and how they worked toward making the necessary changes. They wanted to make their futures different from their lifestyle and choices prior to their incarceration. Participants here also recognized how the experience of incarceration had pushed them to work toward such self-change:

After a bit, it started to change. I started to realize that this isn’t how you want your life to go . . . . (Nate)

I think I was starting to realize that, I wanted more out of life then this, but I didn’t know. Because I only knew one thing [before prison]. (Ryan)

For some, decisions toward self-change started in prison and included being selective about who they associated with in prison and ending some of their pre-prison affiliations, which was not always easy given, as Steve explained, “You do need your friends. Whether you’re a gang or your associates or whatever you want to say, you do need them.” James, who was serving a life sentence in the community, explained how he learned that

Everybody can change when their ready to change. Some guys want to change but their not ready. I think that’s what it is with a lot of younger kids and some people older, yeah they want to do it and they think in their heart or in their mind they want to change and they try but their just their not ready yet . . .

He continued,

I’m fortunate that I had, I am, in a sense doing a life sentence so I had a lot of supportive people there for me, but if I wasn’t doing a life sentence then I would have been . . . I think the system does work if you want it to work, if you want help and you’re ready for help it works, the resources are there but you have to want it and you have to be ready for it.

Like Stewart above who, in saying he “grew up,” demonstrated a degree of maturity that suggests he was then ready and able to make more positive life choices despite the prison environment he found himself within, James recognized the complexities of “changing.” He described such change as being more than a quick decision and instead a long-term process rooted in the cognitive changes that researchers have long associated with desistance more generally (Bottoms et al., 2004; Giordano et al., 2002; Laub and Sampson, 2001; Maruna, 2001). Participants’ narratives signify a deep desire for self-change and transformation. Our findings suggest that imprisonment, by immersing prisoners in a space that forced self-reflection away from the distractions of everyday life, changed the ways prisoners talked about themselves and the sorts of qualities that they believed would help them change their life for the better in the long term.

In addition to space, imprisonment also offered time for self-reflection. More specifically, for participants, imprisonment functioned as a sort of disruption or break from pre-prison time—time that was associated with pain, hurt, and remorse, that could not be recaptured (see also Wahidin, 2006). Prison time, thus, in a sense, created a welcome, albeit forced, opportunity to reflect back and envision time in the future as something that participants would value and use more consciously and purposefully to the benefit of themselves and others. While time in prison is often associated with punishment (i.e. prisoners’ time is strictly regulated and disciplined) and simple survival (i.e. prisoners need to find coping strategies to make it through prison time), our findings suggest that prison is also a temporal break from pre-prison times and as such, in the eyes of prisoners, may function as an opportunity to start time new.

## Discussion: Desistance, positivity, and long-term implications

For participants, prisons, in essence, functioned as a time and space for forced, self-reflection which pushed them to reflect on who they were prior to their incarceration but also who they wanted to be at release—recognizing that who they were prior to incarceration did not necessary reflect who they had become. Findings from this study, we hope, are relevant specifically for scholars studying prison release and re-entry. Scholars have shown that prison experiences affect and shape a person’s re-entry into the community (Petersilia, 1999; Petersilia, 2003). Much of the focus here has been on showing how prisons fail to adequately or “successfully” prepare prisoners for re-entry, and that the lack of in-prison supports and release planning are one of several aggravating factors that shape ex-prisoners’ chances of securing employment, re-establishing social relationships, and re-building their lives after release. In our study, interviewees did report benefiting from prison in a multitude of different ways, focused primarily on learning inner strength and finding new interpretations of their lived experiences as they sought to create a more positive life path for themselves (and by extension, their loved ones). Based on our findings, the following two questions emerge. First, how may these more positive impacts of the prison experience shape people’s lives after release and what can be done to help former prisoners maintain their more positive sense of self as they return from prison to the community? Second, are these benefits, the positive outcomes of prison living, worth the risks, threats, and pains of incarceration?

Starting with the first question, we invite scholars to engage in further empirical work on how experiences of incarceration—positive and negative—shape a person’s re-entry and reintegration into the community. Successful re-entry policies must pay attention to prisoners’ experiences in prison, and more specifically, the ways prisoners make sense of and process the impacts of their imprisonment on their self and persona. Adding to existing conceptualizations of prisons as sites of “importation” and “deprivation” (Irwin and Cressey, 1977; Sykes, 1958), we would argue that prisons should also be conceived of and studied as sites of “exportation” that impact prisoners’ trajectories after release, including their relationships with others. Essential to a person’s successful release is that correctional staff (e.g. parole officers) and other community actors pay attention to and recognize the change in self that prisoners undergo while in prison, and that they appreciate this change as a form of desistance that prisoners, for the most part, have worked to achieve on their own. McNeill (2016) calls this tertiary desistance, referring to the recognition by others that prisoners have changed (see also Weaver, 2013). In addition, for prisoners to capitalize on their inner strength and ensure that qualities such as patience and calmness can be maintained or better strengthened after release, returning prisoners need to be provided with both the time and space to (re-)connect with loved ones; continue to care for their physical and mental health and treatment needs; and contemplate their future. Given what we know about the barriers to re-entry, the stress associated with release, and the amount of time that is often spent in pursuit of finding employment and housing (see, for example, Sugie, 2018), time and space for re-connection and reflection are likely to fall behind ex-prisoners’ immediate and pressing needs. They instead focus on meeting the demands of securing employment and housing, following their conditions of release, and dealing with the distractions and stressors of everyday life. We would argue that the penal system can work to buffer these stressors and provide ex-prisoners with the time and resources needed to hold on to their new sense of self. This can be done, for example, by listening to ex-prisoners’ own priorities in terms of their reintegration and their conceptions of their future (see also Maier 2020), by providing mental health and emotional supports, and by ensuring that material needs (e.g. housing) are taken care of, especially during the early stages of release.

In response to the second question whether some of the reported benefits are worth the pains and destructiveness of prison living, we argue that the simple answer is always no. We come to our conclusion in part because interviewees did not attribute the positive impacts to the prison or the institution; instead, the positives were attributed to self, their belief in self, and their ability to either dig deep and find self or to reinvent, positively, their narrative and future self. In line with Crewe and Ievins (2019), our data also suggest that the more positive accounts of prison can be attributed to the fact that incarceration provides *a space* for self-reflection, for “getting clean,” and for practicing patience and calmness.

At the same time, we would also highlight that we cannot say for certain that the institutional form of the prison does not help people positively develop or change their identity. Rather, the experience of incarceration gave prisoners a chance for self-reflection through which they learnt to manage and navigate prison living (e.g. learning patience) while reflecting on their own persona. We urge future researchers to further examine how exactly the prison environment, which is laced with hostility and pain, informs the identity of prisoners. Is there something about prison/imprisonment that does indeed “help” despite clear experiences of risk and pain? We urge researchers to consider further how the length of time spent in prison or the particular setting of the prison, for example, may impact prisoners’ sense of self so that we can gain a better and more nuanced understanding of what exactly it is about the prison or the process of incarceration that may impact prisoners’ sense of self.

## Concluding remarks: Positivity and punishment

In our reflections, positivity remains relatively understudied among scholars of punishment. In response, we conclude this article by reflecting on some of the possible reasons for the omission of positivity in punishment studies and how studying positivity may change how we think about prisons and punishment. Pointing to the positive experiences of prison feels rather uncomfortable as well as, politically, counter-productive to calls for decarceration and alternatives to incarceration. Studying and acknowledging positivity may be seen as indirectly approving of a person’s incarceration or producing knowledge that can then be mobilized (by politicians, prosecutors, academics, and others) to justify a person’s incarceration as it suggests that prison can have a rehabilitative, healing, or otherwise positive function in people’s lives. Rather than striving toward reducing the use of incarceration, acknowledging positivity may be seen as contributing to the continued or even expanded use of prisons or other forms of punishment. Furthermore, focusing on positivity may risk playing into dominant tropes that prisoners are “defective” or “bad” and in need of self-change, moral reformation, and state intervention as positivity clearly includes a focus on individual change and betterment. Positivity also risks placing blame on and responsibilizing those who, for whatever reason, are not able to find positive self-change (e.g. patience and calmness) in prison.

While we acknowledge such risks and take them seriously, we argue that positivity is an element of incarceration that is worthy of study for those interested in understanding the complex nature and problems of punishment, as well as those focused on studying prisoners’ narratives of desistance. As we have mentioned before, pointing to the positive aspects of a person’s incarceration, or more precisely, the forms of positive change that can happen within prison, does not discount that prison experiences are inherently painful, depriving, and often degrading. Nor does it distract from or call into question the rich and uncontestable evidence of the harms of the prison, for those incarcerated and their families, but also those working in said spaces. We would argue instead that studying positivity helps us understand the ambivalent nature of the prison and the prison experience where narratives of positive self-change can occur *alongside* deeply painful and threatening experiences. Nothing is all bad, and acknowledging positivity is a necessary, albeit uncomfortable, aspect of studying prisons, and by extension punishment, in its complexity and ambivalence. We would also contend that pointing to positive change that can result within the context of incarceration does not imply that imprisonment is *good* for or helpful to people. Rather, what we see in our data is that prisoners, despite the hostility that surrounds them, find narratives of self-change, hope, and resilience (see also O’Donnell, 2014). Given that positivity is one (albeit not the dominant) reality of prison living, we urge scholars of punishment to consider more seriously how to talk about and present prisoners’ narrations of positivity in thoughtful and humanizing ways that show prisoners’ strength and resiliency without glorifying the struggles or harms of imprisonment. By paying attention to the more positive or productive forms of change that can take place in prison, we gain a better and much needed understanding of how prisoners adapt to and navigate their time in prison (see also O’Donnell, 2014). That prisoners appear to find positive narratives of self-change even as they experience hostility and negativity in prison demonstrates clearly that prison adaptations are complex and nuanced, and that on an individual level, prisoners find meaningful ways to adapt to and make sense of their incarceration.

We would also urge scholars to think further about positivity in an effort to consider how positive self-change can be achieved in the *absence* of incarceration—for example, by providing people with a space to recharge from the struggles of everyday life or by providing with the time that is necessary for anyone to be able to reflect, recharge, and focus on one’s life and future. To conclude, by recognizing positivity, we first highlight, in line with other scholars (see, for example, Werth, 2012), that punishment has both repressive and productive elements. Specifically, we show, that for some prisoners, productivity is also a response to some of the more harmful and suffering aspects of incarceration. In this respect, we urge further researchers to examine *how* positive self-change develops in prison to gain a comprehensive and nuanced understanding of how punishment functions and works in the lives of prisoners and the impact it has on people’s trajectories of release.

## References

[bibr1-17488958211031336] Bottoms A Shapland J Costella A , et al. (2004) Toward desistance: Theoretical underpinnings for an empirical study. Howard Journal of Criminal Justice 43(4): 368–389.

[bibr2-17488958211031336] Bucerius M Haggerty KD Dunford TD (2021) Prison as temporary refuge: Amplifying the voices of women detained in prison. The British Journal of Criminology 61(2): 519–537.

[bibr3-17488958211031336] Charmaz K (2006) Constructing Grounded Theory. London: SAGE.

[bibr4-17488958211031336] Crewe B Ievins A (2019) The prison as a reinventive institution. Theoretical Criminology 24: 568–589.

[bibr5-17488958211031336] Giddens A (1991) Modernity and Self-identity: Self and Society in the Late Modern Age. Cambridge: Polity Press.

[bibr6-17488958211031336] Giordano PC Cernkovich SA Rudolph JL (2002) Gender crime and desistance: Toward a theory of cognitive transformation. American Journal of Sociology 107(4): 990–1064.

[bibr7-17488958211031336] Goffman E (1961) Asylums: Essays on the Social Situation of Mental Patients and Other Inmates. New York: Doubleday.

[bibr8-17488958211031336] Hemmens C Marquart J (1999) Straight time: Inmates’ perceptions of violence and victimization in the prison environment. Journal of Offender Rehabilitation 28(3): 1–21.

[bibr9-17488958211031336] Irwin J Cressey DR (1977) Thieves, convicts, and the inmate culture. In: Legger RG Stratton JR (eds) The Sociology of Corrections. New York: John Wiley and Sons, pp. 133–147.

[bibr10-17488958211031336] Kellar M Wang H-M (2005) Inmate assaults in Texas county jails. The Prison Journal 85(4): 515–534.

[bibr11-17488958211031336] Laub J Sampson R (2001) Understanding desistance from crime. Crime and Justice 28: 1–69.

[bibr12-17488958211031336] Liebling A Laws B Lieber E , et al. (2019) Are hope and possibility achievable in prison? The Howard Journal of Crime and Justice 58(1): 104–126.

[bibr13-17488958211031336] Maier K (2020) ‘Mobilizing’ prisoner reentry research: Halfway houses and the spatial-temporal dynamics of prison release. Theoretical Criminology. Epub ahead of print 11 January. DOI: 10.1177/1362480619896371.

[bibr14-17488958211031336] Maier KH Ricciardelli R (2018) The prisoner’s dilemma: How male prisoners experience and respond to penal threat while incarcerated. Punishment & Society 21: 231–250.

[bibr15-17488958211031336] Maruna S (2001) Making Good: How Ex-convicts Reform and Rebuild Their Lives. Washington, DC: American Psychological Association.

[bibr16-17488958211031336] Maruna S Wilson L Curran K (2006) Why God is often found behind bars: Prison conversions and the crisis of self-narrative. Research in Human Development 3(2–3): 161–184.

[bibr17-17488958211031336] O’Donnell I (2014) Prisoners, Solitude, and Time. Oxford: Oxford University Press.

[bibr18-17488958211031336] McNeill F (2016) Desistance and criminal justice in Scotland. In: Croall H Mooney G Munro R (eds) Crime, Justice and Society in Scotland. London: Routledge, pp. 200–216.

[bibr19-17488958211031336] Petersilia J (1999) Parole and prisoner reentry in the United States. Crime and Justice 26: 479–529.

[bibr20-17488958211031336] Petersilia J (2003) When Prisoners Come Home. Oxford: Oxford University.

[bibr21-17488958211031336] Ricciardelli R (2014) Surviving Incarceration: Inside Canadian Prisons. Waterloo, ON: Wilfrid Laurier University Press.

[bibr22-17488958211031336] Ricciardelli R Clow KA White P (2010) Masculinity portrayals in men’s lifestyle magazines. Sex Roles: A Journal of Research 63(1): 64–78.

[bibr23-17488958211031336] Schinkel M (2014) Legitimacy and the Impact of the Prison Environment. In: Schinkel M (ed.) Being Imprisoned. New York: Springer, pp. 62–94.

[bibr24-17488958211031336] Scott S (2010) Revisiting the total institution: Performative regulation in the reinventive institution. Sociology 44(2): 213–231.

[bibr25-17488958211031336] Strauss A Corbin J (1990) Basics of Qualitative Research: Grounded Theory Procedures and Techniques. Newbury Park, CA: SAGE.

[bibr26-17488958211031336] Sugie NF (2018) Work as foraging: A smartphone study of job search and employment after prison. The American Journal of Sociology 123(5): 1453–1491.

[bibr27-17488958211031336] Sykes GM (1958) The Society of Captives. Princeton, NJ: Princeton University Press.

[bibr28-17488958211031336] van Ginneken EF (2014) Interviewing in Prison: Understanding the Impact of Imprisonment. New York: SAGE.

[bibr29-17488958211031336] Wahidin A (2006) Time and the Prison Experience. Sociological Research Online 11(1): 1–10.

[bibr30-17488958211031336] Weaver B (2013) Desistance, reflexivity and relationality: A case study. European Journal of Probation 5(3): 71–88.

[bibr31-17488958211031336] Werth R (2012) I do what I’m told, sort of: Reformed subjects, unruly citizens, and parole. Theoretical Criminology 19(3): 329–346.

[bibr32-17488958211031336] Wolff N Shi J (2001) Patterns of victimization and feelings of safety inside prison: The experience of male and female inmates. Crime & Delinquency 57(1): 29–55.

[bibr33-17488958211031336] Wolff N Shi J Siegel JA (2009) Patterns of Victimization Among Male and Female Inmates: Evidence of an Enduring Legacy. Violence and Victims 24(4): 469–484. 19694352 10.1891/0886-6708.24.4.469 PMC3793850

[bibr34-17488958211031336] Wolff N Shi J Blitz CL , et al. (2007) Understanding Sexual Victimization Inside Prisons: Factors that Predict Risk. Criminology & Public Policy 6(3): 535–564.

